# Granzyme B Degraded Type IV Collagen Products in Serum Identify Melanoma Patients Responding to Immune Checkpoint Blockade

**DOI:** 10.3390/cancers12102786

**Published:** 2020-09-28

**Authors:** Christina Jensen, Dovile Sinkeviciute, Daniel Hargbøl Madsen, Patrik Önnerfjord, Morten Hansen, Henrik Schmidt, Morten Asser Karsdal, Inge Marie Svane, Nicholas Willumsen

**Affiliations:** 1 Biomarkers & Research, Nordic Bioscience, 2730 Herlev, Denmark; dsi@nordicbio.com (D.S.); mk@nordicbio.com (M.A.K.); nwi@nordicbio.com (N.W.); 2 Biotech Research & Innovation Centre (BRIC), University of Copenhagen, 2200 Copenhagen, Denmark; 3 Department of Clinical Sciences Lund, Lund University, 221 84 Lund, Sweden; patrik.onnerfjord@med.lu.se; 4 National Center for Cancer Immune Therapy (CCIT-DK), Department of Oncology, Copenhagen University Hospital, 2730 Herlev, Denmark; daniel.hargboel.madsen@regionh.dk (D.H.M.); morten.hansen.01@regionh.dk (M.H.); inge.marie.svane@regionh.dk (I.M.S.); 5 Department of Oncology, Aarhus University Hospital, 8200 Aarhus, Denmark; henrschm@rm.dk

**Keywords:** tumor microenvironment, extracellular matrix, collagen, fibrosis, T-cell infiltration, biomarker, immunotherapy, immune checkpoint inhibitor, ipilimumab, melanoma

## Abstract

**Simple Summary:**

Novel biomarkers that can identify melanoma patients responding to immune checkpoint inhibitor therapy are urgently needed. As high T-cell infiltration and low fibrotic activity are associated with response, we aimed to examine the serum biomarker potential of granzyme B degraded type IV collagen (C4G) products in combination with the fibrosis biomarker PRO-C3. We found that high C4G combined with low PRO-C3 has the potential to identify patients responding to immune checkpoint inhibitor therapy suggesting that these biomarkers may provide a non-invasive tool for patient selection and therapeutic decision-making in the future.

**Abstract:**

A T-cell permissive tumor microenvironment, characterized by the presence of activated T cells and low fibrotic activity is crucial for response to immune checkpoint inhibitors (ICIs). Granzyme B has been shown to promote T-cell migration through the basement membrane by the degradation of type IV collagen. In this study, we evaluated the biomarker potential of measuring granzyme B-mediated degradation of type IV collagen (C4G) in combination with a fibroblast activation biomarker (PRO-C3) non-invasively for identifying metastatic melanoma patients responding to the ICI ipilimumab. A monoclonal antibody was generated against C4G and used to develop a competitive electro-chemiluminescence immunoassay. C4G and PRO-C3 were measured in pretreatment serum from metastatic melanoma patients (*n* = 54). The C4G assay was found specific for a granzyme B-generated neo-epitope on type IV collagen. The objective response rate (ORR) was 2.6-fold higher (18% vs. 7%) in patients with high C4G levels (>25th percentile) vs. low levels (≤25th percentile). Likewise, high C4G levels at baseline were associated with longer overall survival (OS) (log-rank, *p* = 0.040, and hazard ratio (HR) = 0.48, 95%CI: 0.24–0.98, *p* = 0.045). Combining high C4G with low PRO-C3 correlated with improved OS with a median OS of 796 days vs. 273 days (*p* = 0.0003) and an HR of 0.30 (95%CI: 0.15–0.60, *p* = 0.0006). In conclusion, these results suggest that high granzyme B degraded type IV collagen (C4G) combined with low PRO-C3 quantified non-invasively has the potential to identify the responders to ICI therapy.

## 1. Introduction

Immune checkpoint inhibitors (ICIs) brought a paradigm shift in the treatment of patients with metastatic melanoma by offering an opportunity for durable responses [1]. However, despite the clinical success of anti-cytotoxic T lymphocyte antigen 4 (CTLA-4), anti-programmed cell death protein 1 (PD-1), and anti-PD-1 ligand (PD-L1), only a subset of cancer patients experiences a long-term survival benefit. Biomarkers related to the efficacy of treatment and mechanism of resistance can guide patient selection and treatment decisions to improve clinical outcomes, avoid severe toxicity in patients not likely to respond, and assist in guiding the spending of health care resources.

ICIs reactivate the T cells to attack the cancer cells, and tumor-infiltrating T cells are therefore crucial for clinical efficacy and good prognosis. Clinical responses occur most often in patients with a pre-existing T-cell infiltrate and anti-tumor response that may have been blocked through checkpoint signaling [2]. Advanced melanoma patients that respond to anti-CTLA-4 therapy have higher levels of circulating memory CD4+ and CD8+ T cells at baseline [3]. In addition, patients responding to ICIs have high pre-treatment levels of CD4+ and CD8+ T cells in the tumor parenchyma in proximity to the tumor cells, a patient profile defined as an immune-inflamed phenotype (“hot” tumor) [2,4,5]. On the other hand, patients with no or few T cells in either the tumor parenchyma or surrounding stroma, defined as an immune-desert phenotype (“cold” tumor), rarely respond to ICI therapy.

In recent years, a tumor-specific extracellular matrix (ECM) with increased collagen density and stiffness, so-called desmoplasia, has emerged as a key factor that influences response to ICI treatment. Excessive collagen deposition affects the location and migration of T cells and associates with resistance to immunotherapy [6,7,8,9,10,11]. Lack of response occurs when the cytotoxic T cells are excluded from the tumor and instead trapped in the fibroblast- and collagen-rich peritumoral stroma, a patient profile defined as an immune-excluded phenotype [5,10]. In addition, a recent study showed that a high-density collagen matrix directly reduces T-cell proliferation and cytotoxic activity, suggesting an immunosuppressive mechanism of dense collagen [12]. We have previously shown that the non-invasive neo-epitope biomarker PRO-C3 reflecting fibrotic activity and type III collagen deposition identifies melanoma patients with poor response to ICI therapy (anti-CTLA-4) [13].

Identifying non-invasive predictive biomarkers related to a T-cell permissive tumor microenvironment without a dense stroma barrier would provide a precision medicine tool for patient selection and therapeutic decision making. Interestingly, T cells express proteases that induce invasive behavior [14,15]. Cytotoxic T cells release the serine protease granzyme B to break down type IV collagen to pass through basement membranes on the route from the blood into the tumor tissue [14]. Furthermore, granzyme B has a prominent cytotoxic role in mediating cancer cell death, an anti-tumor activity that correlates with response to ICI therapy [2,16].

In this study, we hypothesized that specific granzyme B degraded type IV collagen fragments are released to the circulation and may have a biomarker potential of identifying cancer patients that respond to ICI therapy. In addition, as fibrotic activity and a dense collagen matrix influence the clinical response, we hypothesized that combining this novel biomarker measuring a granzyme B-generated neo-epitope on type IV collagen with the fibrosis biomarker PRO-C3 would have additional predictive value. To investigate this, we developed and validated a competitive electro-chemiluminescence immunoassay (ECLIA) targeting a granzyme B-generated neo-epitope on type IV collagen degradation fragments (C4G) and evaluated its biomarker potential for identifying metastatic melanoma patients responding to the anti-CTLA-4 antibody ipilimumab, both alone and in combination with the fibrosis biomarker PRO-C3. Our data shows that granzyme B degraded type IV collagen (C4G) associates with favorable anti-CTLA-4 treatment response in metastatic melanoma patients. Furthermore, when combining high C4G with low PRO-C3 (tumor fibrosis), patients with this special phenotype have an even better chance of responding compared to high C4G levels alone.

## 2. Results

### 2.1. Specificity of the C4G Assay

**Figure 1:**
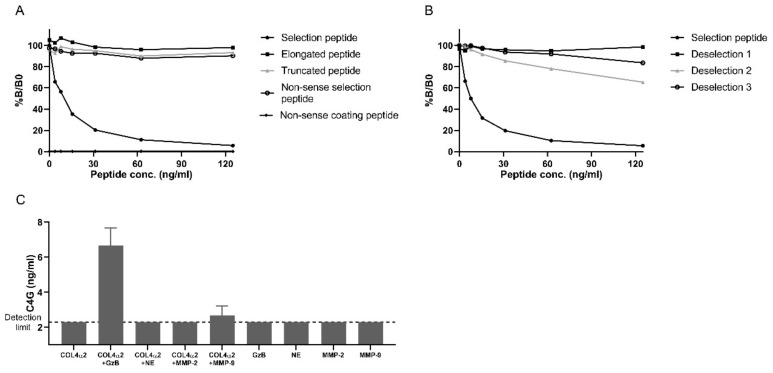
Specificity of the C4G monoclonal antibody. The monoclonal antibody’s reactivity in the competitive C4G ECLIA was tested toward (**A**) the selection peptide (MGNTGPTGAV), an elongated peptide (FMGNTGPTGAV), a truncated peptide (GNTGPTGAV), a non-sense selection peptide (LLARDFEKNY), and a non-sense coating peptide (LLARDFEKNY-K-biotin) and (**B**) the selection peptide (MGNTGPTGAV) and the deselections peptide 1 (MGQTGPTGAV), 2 (MGNSGPTGAV), and 3 (QGNTGPTGAV). %B/B0: B equals the intensity of a sample well (OD at x ng/mL peptide) and B0 equals the maximum intensity (OD at 0 ng/mL peptide). (**C**) The α2 chain of type IV collagen was incubated for 24 h without or with the serine proteases granzyme B (GzB) or neutrophil elastase (NE), or the matrix metalloproteinases (MMPs) MMP-2 or MMP-9. The buffer added proteases alone were included as negative controls. Data is based on two independent experiments and presented with mean and standard deviation. The dotted line represents the lower limit of measurement.

To evaluate the new competitive ECLIA targeting a granzyme B-generated neo-epitope on type IV collagen (C4G), the specificity of the monoclonal antibody was tested. The selection peptide, with a sequence unique for human type IV collagen, inhibited the signal in a dose-dependent manner whereas the elongated, truncated, and non-sense selection peptide did not inhibit the signal (Figure 1A). No signal was observed when using a non-sense biotinylated coating peptide (Figure 1A). When the reactivity was tested toward peptides with only one amino acid mismatch compared to the selection peptide, only relative high concentrations of peptide resulted in minor reactivity with a B/B0 ~80% (Figure 1B). Together, these data suggest that the monoclonal antibody is highly specific to the neo-epitope on the selection peptide.

### 2.2. The C4G Assay Detects Granzyme B-Mediated Type IV Collagen Degradation

Next, we investigated the ability of the serine protease granzyme B to degrade type IV collagen and compared it to the serine protease neutrophil elastase and the matrix metalloproteinases (MMPs) MMP-2 and MMP-9. The proteases were incubated with a type IV collagen alpha 2 chain for 1, 4, 24, and 72 h. As shown in Figure 1C, granzyme B mediated high levels of degraded type IV collagen (C4G) after 24 h. Conversely, neutrophil elastase and MMP-2 did not cleave type IV collagen, and MMP-9 mediated only low levels of degraded type IV collagen. Similar results were observed after 72 h. Furthermore, C4G was not detectable in undigested samples (without protease). Altogether, these results indicate that the antibody is specific for a granzyme B-generated neo-epitope on type IV collagen.

### 2.3. The C4G ECLIA Is a Technically Robust Assay

The technical performance of the C4G ECLIA was further assessed through the different technical validation steps summarized in Table 1. The IC50 was 8.8 ng/mL. The lower limit of the measurement range was 2.3 ng/mL, and the upper limit of the measurement range was 788 ng/mL when samples were measured undiluted. The intra- and inter-assay variations were 6% and 8% and below the acceptance criterion of 10% and 15%, respectively. Linearity was detected from undiluted to a 1:4 dilution with dilution recoveries at 94% and 106% for serum and EDTA plasma, respectively. Spiking recovery of serum in serum resulted in a mean recovery of 102%. After 4 freeze/thaw cycles, the analyte recovery in serum was 96%. After prolonged storage of human serum at 4 °C or 20 °C for 48 h, the analyte recoveries were 122% and 109%, respectively. No interference was detected from low or high contents of lipemia or hemoglobin in serum with recoveries ranging from 92–111%. Low content of biotin did not interfere with the analyte whereas high content of biotin did with recoveries at 94% and 71%, respectively. The acceptance criterion of the recoveries was within 100% ± 20%. Together, these results indicate that the C4G ECLIA is a technically robust assay.

### 2.4. Patients Characteristics

To evaluate the biomarker potential of C4G, we measured baseline serum samples from 54 metastatic melanoma patients treated with ipilimumab. Baseline patient characteristics are shown in Table 2. The patients had a median age of 68 years, 57% of the patients were females, 45% had received prior systematic therapy, and 21% had elevated lactate dehydrogenase (LDH) levels (≥250 IU/L). Of the 54 patients, 37 patients were deceased within the follow-up period. The median overall survival (OS) was 410 days (range: 40–1133 days). According to Response Evaluation Criteria in Solid Tumors (RECIST), 1 patient had a complete response (CR), 7 patients had a partial response (PR), 11 patients had stable disease (SD), and 35 patients had progressive disease (PD).

### 2.5. High C4G Levels at Baseline Associate with Good Clinical Response to Ipilimumab

**Figure 2:**
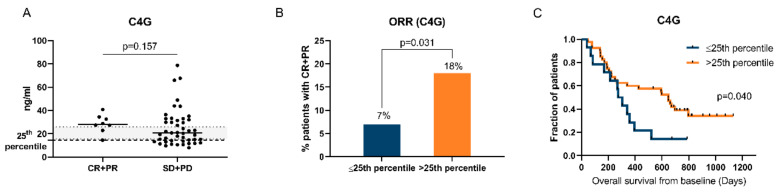
High C4G levels at baseline associate with good clinical response to ipilimumab. (**A**) C4G levels at baseline in serum from metastatic melanoma patients treated with ipilimumab with complete response (CR) and partial response (PR) (*n* = 8) were compared to C4G levels in patients with stable disease (SD) and progressive disease (PD) (*n* = 46) with a Mann–Whitney test. The black lines represent the median value, and the black dotted line represents the 25th percentile cut-point. The reference limit for healthy individuals is illustrated by the gray area (mean = 20.7 ng/mL, 95%CI = 15.5–25.9 ng/mL). (**B**) The objective response rate (ORR) in patients with low C4G levels (≤25th percentile) (*n* = 14) was compared to patients with high C4G levels (>25th percentile) (*n* = 40) with Fisher’s exact test. (**C**) Overall survival for the same groups of patients was analyzed by Kaplan–Meier curves and a log-rank test. A *p*-value of *p* < 0.05 was considered statistically significant.

We first assessed the association between C4G levels and the objective response rate (ORR). C4G was slightly elevated in patients with CR and PR (28.0 ng/mL) compared to patients with SD and PD (20.6 ng/mL) (*p* = 0.157) (Figure 2A), although no statistically significant difference was found. Most of the patients with CR and PR had C4G levels above the reference limits of healthy individuals whereas the patients with SD and PD had C4G levels below, within, or above the reference limits. As the majority of responding patients (CR + PR) had C4G levels above the 25th percentile, the C4G biomarker levels were dichotomized by the 25th percentile cut-point. The ORR was 2.6-fold higher (18% vs. 7%) in patients with high C4G levels (>25th percentile) compared to low levels (≤25th percentile) (*p* = 0.031) (Figure 2B).

Next, we evaluated the association between high C4G levels and OS. Using the Kaplan–Meier method, high C4G levels (>25th percentile) were significantly associated with increased OS compared to low levels (≤25th percentile) (*p* = 0.040) (Figure 2C). The median OS was 646 days in biomarker-high patients compared to 290 days in biomarker-low patients.

The ability of C4G to predict OS was then investigated with the Cox proportional-hazards model (Table 3). Using the univariate Cox regression analysis, high C4G was found to be predictive of a 52% reduced risk of dying compared to low C4G (hazard ratio (HR) = 0.48, 95% confidence interval (CI) = 0.24–0.98, *p* = 0.045). The independent predictive value of C4G was then evaluated by the multivariate Cox regression analysis. When C4G was adjusted for the covariates age, high LDH (≥250 IU/L), and prior systematic therapy, high C4G remained borderline predictive of OS with a reduced risk of dying of 57% (HR = 0.43, 95%CI = 0.19–1.00, *p* = 0.051). Of the other covariates included in the multivariate analysis, high LDH (≥250 IU/L) predicted poor OS (HR = 2.50, 95%CI = 1.20–5.21, *p* = 0.015).

### 2.6. Patients with High C4G and Low PRO-C3 Levels Have a Better Chance of Responding

**Figure 3:**
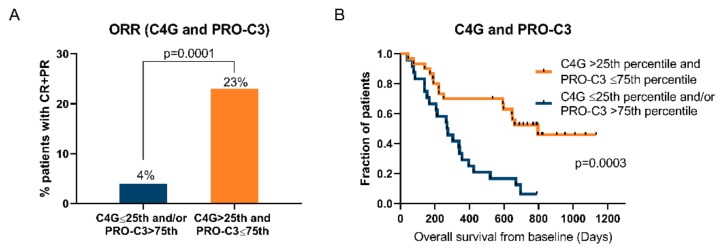
Patients with high C4G and low PRO-C3 levels have a better chance of responding. (**A**) The objective response rate (ORR) in metastatic melanoma patients with high C4G (>25th percentile) and low PRO-C3 (≤75th percentile) levels at baseline were compared to patients with low C4G and/or high PRO-C3 levels with Fisher’s exact test. (**B**) Overall survival for the same groups of patients was analyzed by Kaplan–Meier curves and a log-rank test. A *p*-value of *p* < 0.05 was considered statistically significant.

Next, we investigated if combining the fibrosis biomarker PRO-C3 with C4G could provide additional clinical value. We have previously seen that low PRO-C3 levels (≤75th percentile) were associated with improved clinical response in these metastatic melanoma patients treated with ipilimumab [13]. When assessing response according to RECIST criteria, the ORR was 5.8-fold higher (23% vs. 4%) in patients with both high C4G (>25th percentile) and low PRO-C3 levels (≤75th percentile) compared to patients with low C4G (≤25th percentile) and/or high PRO-C3 (>75th percentile) (*p* = 0.0001) (Figure 3A). Interestingly, all the patients with CR and PR had low PRO-C3 levels (≤75th percentile). Furthermore, 33% of the patients with SD and PD who had high C4G levels (>25th percentile) (Figure 2A) also had high PRO-C3 levels (>75th percentile), including the three patients with the highest C4G levels (range: 65.9–78.6 ng/mL).

When evaluating survival outcomes by Kaplan–Meier, patients presenting with both high C4G (>25th percentile) and low PRO-C3 (≤75th percentile) levels had longer OS compared to patients with low C4G (≤25th percentile) and/or high PRO-C3 (>75th percentile) (*p* = 0.0003) (Figure 3B). The median OS was 796 days in patients with high C4G and low PRO-C3 compared to 273 days in the rest of the patients. In addition, univariate Cox regression identified this biomarker combination with high C4G and low PRO-C3 as predictive of a 70% reduced risk of dying (HR = 0.30, 95%CI = 0.15–0.60, *p* = 0.0006), and the predictive value remained significant when adjusted for age, high LDH, and prior systematic therapy (HR = 0.35, 95%CI = 0.18–072, *p* = 0.004) (Table 3).

## 3. Discussion

Identification of predictive biomarkers for immunotherapies is challenging because complex and heterogenic factors from the tumor, host, and environment affect tumor immunity [5].

In this study, we showed the biomarker potential of specific collagen remodeling products for identifying patients responding to ICI therapy. By developing a technically robust assay, we identified and quantified granzyme B-mediated type IV collagen degradation fragments (C4G) in the circulation of metastatic melanoma patients. High C4G levels at baseline were associated with good clinical response to anti-CTLA-4 therapy both when assessing ORR and OS. By combining high C4G with low PRO-C3, a biomarker of type III collagen formation (fibrosis), metastatic melanoma patients with this phenotype had an even better chance of responding compared to high C4G levels alone. To our knowledge, this is the first study to show that specific granzyme B degraded type IV collagen fragments (C4G) have biomarker potential in metastatic melanoma patients treated with ICI therapy.

An important finding related to the protease-specific processing of the matrix and relevant to the neo-epitope technology is the fact that high pre-treatment levels of the ECM-derived biomarker C4M, measuring MMP degraded type IV collagen, associate with poor survival outcomes in these metastatic melanoma patients receiving ipilimumab [13]. MMP-mediated collagen degradation is associated with tumor activity and metastatic dissemination [17,18,19]. C4G and C4M both measure a neo-epitope on type IV collagen but at two different sites generated by two different proteases. These findings indicate that different protease profiles may dominate in distinct tumor types or pathological events and support the value of measuring pathological specific neo-epitopes and not just the total protein [20,21].

A high pre-treatment T-cell infiltrate associates with a response to ICIs [3], and we observed high C4G baseline levels (>25th percentile) in responding patients. The C4G assay is specific for granzyme B-mediated degradation of type IV collagen and not for neutrophil elastase or MMP degradation, supporting that C4G reflects granzyme B activity and granzyme B-induced processes. These results fit a scenario where granzyme B degraded type IV collagen fragments (C4G) are released to the circulation when cytotoxic T cells release granzyme B to break down type IV collagen to pass through basement membranes on the route from the blood into the tumor tissue [14]. This link between T-cell infiltration and C4G levels should be investigated in future studies, for example by CD8+ T-cell infiltration scores and tumor-infiltrating lymphocyte assessments.

**Figure 4:**
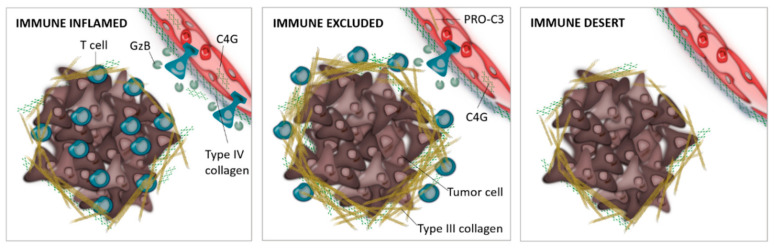
Hypothetical illustration showing how the ECM-derived biomarkers may associate with cancer-immune phenotypes. (**Left**) During T-cell infiltration into the tumor microenvironment, T cells release granzyme B (GzB) to break down type IV collagen to pass through the basement membrane which may result in released fragments of granzyme B degraded type IV collagen (C4G) into the circulation. In the immune-inflamed phenotype, many of these T cells are positioned in the tumor parenchyma, while in the immune-excluded phenotype (**Middle**), the T cells are retained in the high-density collagen barrier that may release type III collagen fragments into the circulation (PRO-C3). (**Right**) In the immune-desert phenotype, few or no T cells are found in the tumor microenvironment.

In this study, a subset of the non-responding patients had high C4G levels which could have questioned the biomarker potential of C4G. However, interestingly, a subgroup of those patients, also had high levels of the fibrosis biomarker PRO-C3, suggesting and supporting that a high-density collagen matrix affects the T-cell ability to kill the tumor cells and hereby impact response and outcome of patients. These results also suggest that PRO-C3 is a necessary second biomarker to C4G as both fibrosis and T-cell activity influence the response to ICI therapy. It could be speculated that these patients had an immune-excluded phenotype, where infiltrating T cells were excluded from the tumor parenchyma by a dense collagen stroma, resulting in a blockade of anti-tumor activity and reduced response. This hypothetical explanation is illustrated in Figure 4. Moreover, patients with high C4G levels and low PRO-C3 levels had better survival outcomes compared to C4G and PRO-C3 alone [13], suggesting that these patients may have an immune-inflamed phenotype that correlates with clinical response (Figure 4). There is a major medical need for circulating ICI biomarkers, and although the associations between the specific collagen turnover biomarkers and immune profiles are hypothetical and require further studies, the C4G and PRO-C3 biomarkers show potential for predicting response to ICI therapy.

The link between type III collagen deposition (PRO-C3) and resistance to ICIs is supported by a study of ECM gene dysregulation, in which a set of transforming growth factor-beta-(TGF-β)-driven ECM genes, including COL3A, predict ICI failure independent of tumor type [9]. In addition, COL3A was found to be upregulated in T-cell-excluded tumors in hepatocellular carcinoma patients, supporting that collagens act as a barrier that blocks cytotoxic T-cell infiltration [22]. The immune regulatory receptor leukocyte-associated immunoglobulin-like receptor 1 (LAIR-1) expressed on PBMCs has two binding sites for type III collagen, suggesting a direct immune regulating role of type III collagen [23]. High collagen density reduces cytotoxic T-cell activity [12] and could be proposed as one of many strategies that tumors develop to escape anti-tumor immune responses. Furthermore, a recent study illustrates that combined inhibition of TGF-β signaling and the PD-L1 checkpoint enables T-cell infiltration into the tumors, resulting in anti-tumor activity and tumor shrinkage [10]. The C4G and PRO-C3 biomarker combination may provide a non-invasive precision medicine tool for identifying this patient subgroup with an immune-excluded phenotype that may benefit from anti-TGF-β and ICI combination therapy.

Expression of PD-L1, tumor mutation burden, cytokine secretion, tumor-infiltrating T cells, B cell infiltrates in the form of tertiary lymphoid structures in tumors, blood immune cells, and gut microbiota have been proposed as predictive biomarkers in the ICI setting [24,25,26,27,28,29]. However, despite the association with clinical response, the discovery of a single predictive biomarker appears unachievable and an algorithm with different tumor microenvironment components is probably the road ahead. In this study, an additive predictive value was achieved when combining C4G and PRO-C3. While some of the current biomarkers require a tumor tissue biopsy for assessment limited by tumor heterogenicity and difficulties in obtaining fresh tumor material, the collagen remodeling biomarkers C4G and PRO-C3 are measured non-invasively in a liquid biopsy, with the advantages of being homogeneous and easy to obtain.

The clinical validation of the C4G and PRO-C3 biomarkers is limited by the small patient cohort size and that almost half of the patients received systemic treatment prior to the anti-CTLA-4 therapy. Thus, it is desirable to validate these findings in larger patient cohorts that are treated first-line. The predictive biomarker potential of C4G and PRO-C3 should be validated in larger prospective randomized trials that include different kinds of treatment. In addition, the cut-point discriminating high and low biomarker levels should be established and validated further in future studies. Given the emerging indications of ECM having multiple roles in regulating the cancer-immunity cycle [30,31], C4G and PRO-C3 may also have a predictive potential in melanoma and other cancer types treated with anti-PD-1 or anti-PD-L1 therapy. It is a limitation that the C4G and PRO-C3 biomarkers have not been assessed in tumor samples, e.g., with microscopy, and it could be valuable to correlate the serological biomarker levels of PRO-C3 and C4G, and PRO-C3 and C4G staining, with tumor measurements of collagen density and T-cell infiltration. The presently shown predictive value of C4G and PRO-C3 provides knowledge of collagen being an essential factor in immuno-oncology and suggests that these non-invasive biomarkers have a potential for guiding precision medicine in the future.

## 4. Materials and Methods

### 4.1. Development of C4G ECLIA

#### 4.1.1. Reagents

All reagents used for the experiments were standard chemicals from Merck (Whitehouse Station, NJ, USA) and Sigma-Aldrich (St. Louis, MO, USA) unless otherwise stated.

#### 4.1.2. Granzyme B Mediated In Vitro Cleavage of Type IV Collagen

Type IV collagen from the human placenta (Sigma Aldrich, cat. no. C5533) was reconstituted to a final concentration of 100 μg/mL in cleavage buffer (50 mM Tris, 150 mM NaCl, pH 7.5). Granzyme B (Abcam, Cambridge, UK, cat. no. ab168093) was added 1:10 (2.5 μg GzB and 25 μg type IV collagen). Digestion of carboxymethylated transferrin with granzyme B was included as a positive control, and cleavage buffer with added granzyme B alone was included as a negative control. The solutions were incubated at 37 °C for 24 and 72 h and then stored at −80 °C until analysis. The activity of granzyme B was confirmed by silver staining according to the manufacturer’s instructions (SilverXpress, Invitrogen, Waltham, MA, USA, cat. no. LC6100) and Coomassie blue staining.

#### 4.1.3. Peptide Identification by Mass Spectrometry

One µg of the sample (corresponding to digested or undigested collagen in 100 µl 50 mM Tris, 150 mM NaCl, pH 7.5 buffer) was reduced by 10 mM dithiothreitol for 30 min at 56 °C and alkylated by 40 mM iodoacetamide for 60 min in the dark at room temperature. Any remaining iodoacetamide was quenched by 10 mM dithiothreitol for 5 min at room temperature. Samples were digested with Lys-C at 1:20 enzyme:substrate ratio (Wako chemicals, Neuss, Germany, cat#125-05061) for 16 h on a shaker at 37 °C. After the addition of 100 μL 1 M NaCl in 1% formic acid, digests were run through 30 kDa filters (PALL Life Sciences, New York, NY, USA, cat#OD030C34) to remove glycosaminoglycans and then desalted with reversed-phase Vydac UltraMicro Spin C18 columns (Harvard Apparatus, Holliston, MA, USA, cat#74-7206) according to the manufacturer’s instructions. Mass spectrometry (MS) analysis was performed on a quadrupole Orbitrap benchtop mass spectrometer, QExactive, (Thermo Scientific, Waltham, MA, USA) equipped with an Easy nano-LC 1000 system (ThermoFisher Scientific, Waltham, MA, USA). Separation was performed on 75 μm × 25 cm, Acclaim Pepmap™ RSLC C18 capillary columns packed with 2 μm particles (ThermoFisher Scientific). The on-line reversed-phase separation was performed using a flow rate of 300 nL/min and a linear binary gradient for 85 min [32]. A spray voltage of + 2000 V was used with a heated ion transfer setting of 275 °C for desolvation. An MS scan (400–1200 *m*/*z*) was recorded in the Orbitrap mass analyzer set at a resolution of 70,000 at 200 *m*/*z*, 1 × 10^6^ automatic gain control target, and 100 ms maximum ion injection time. The MS was followed by data-dependent acquisition with MS/MS scans at a resolution of 17,500 on the 15 most intense multiply charged ions at 2 × 10^4^ intensity threshold with an isolation width of 2 *m*/*z*. Identification was performed using the *Homo sapiens* proteome (UniProt proteome ID UP000005640) with Proteome Discoverer 2.1 software (ThermoFisher Scientific). The processing workflow consisted of the following nodes: Spectrum Selector for spectra pre-processing (precursor mass range: 300–30,000 Da; S/N Threshold: 1.5), Sequest-HT search engine (Protein Database: see above; Enzyme: Lys-C (semi); Max. missed cleavage sites: 2; Peptide length range 6–144 amino acids; Precursor mass tolerance: 10 ppm; Fragment mass tolerance: 0.02 Da; Static modification: cysteine carbamidomethylation; and Percolator for peptide validation (FDR <1% based on peptide q-value). Peptide intensities were quantified using a proprietary algorithm developed in Proteome Discoverer 2.1 (ThermoFisher Scientific).

#### 4.1.4. Selection of Peptides

C-terminal lysine cleavage was noted as Lys-C cleavages and not considered for biomarker selection. The first six amino acids from the N-terminal and C-terminal of each peptide from type IV collagen identified by MS were regarded as a granzyme B-generated neo-epitope besides the Lys-C cleavages. The granzyme B-generated sequences were analyzed for homology to other human proteins and species using the NPS@: Network Protein Sequence Analysis with the Uniprot/Swiss-Prot database [33]. The amino acid sequence ^1355^MGNTGPTGAV^1364^ C-terminal from the cleavage site F^1354^↓M^1355^ was found unique for human type IV collagen α2 chain and selected as the target for antibody production. Synthetic peptides used for monoclonal antibody production and technical evaluation of the ECLIA measuring granzyme B-mediated degradation of type IV collagen (C4G) were purchased from Genscript. The target sequence was used as the selection peptide (MGNTGPTGAV). The immunogenic peptide (MGNTGPTGAV-GGC-KLH) was generated by covalently linking the selection peptide to Keyhole Limpet Hemocyanin (KLH) carrier protein with the addition of glycine and cysteine residues in between to ensure right linking. A biotinylated peptide (MGNTGPTAV-K-biotin) was used as a coating peptide. As several other proteins have a similar amino acid sequence as the target sequence, but with a different amino acid at positions 1, 3, or 4 from the N-terminal, they were included in the specificity test of the antibody.

#### 4.1.5. Monoclonal Antibody Production and Clone Characterization

Female Balb/C mice of 6–7 weeks of age were immunized subcutaneously with 200 μL emulsified antigen containing 100 µg immunogenic peptide (MGNTGPTGAV-GGC-KLH) with Stimune Immunogenic adjuvant (ThermoFisher, cat. no. 7925000) repeatedly every second week until stable titer levels were obtained. A serum titration test was performed to monitor the immune response of the mice by screening serum for reactivity against the selection peptide in a preliminary competitive enzyme-linked immunosorbent assay (ELISA) using biotinylated coating peptide on streptavidin-coated microtiter plates (Roche, Basel, Switzerland, cat. no. 11940279) (Supplementary Figure S1). The mouse with the highest antibody titer rested for four weeks and was then boosted intraperitoneally with immunogenic peptide. After three days, splenocytes were isolated and fused with murine SP2/0 myeloma cells to produce hybridoma cells as previously described [34]. The hybridoma cells were cultured in 96-well microtiter plates, and limited dilution was used to secure monoclonal growth. Supernatants from the monoclonal antibody-producing hybridoma cells were screened for reactivity against the selection peptide in a preliminary competitive ELISA. A monoclonal antibody with reactivity only toward the specific epitope on the selection peptide was successfully identified (Supplementary Figure S2). Next, the clone was purified using protein G columns according to the manufacturer’s instructions (GE Healthcare Life Sciences, Marlborough, MA, USA, cat. no. 17-0404-01).

#### 4.1.6. C4G Assay Protocol

During assay development, an optimal incubation buffer, time, temperature, and concentrations of antibody and coating peptide were determined and the finalized competitive C4G ECLIA protocol was as follows: A MSD GOLD 96-well streptavidin pre-coated plate (Meso Scale Discovery, Rockville, MD, USA, cat. no. L15SA-1) was incubated with 150 μL/well blocking buffer (10 mM phosphate-buffered saline (PBS) with bovine serum albumin (BSA) (5% *w*/*v*) and bronidox (0.36% *v*/*v*), 8 g/L NaCl, pH 7.4) for 60 min at 20 °C with shaking (300 rpm) in darkness. The plate was coated with 25 μL/well biotinylated coating peptide dissolved in assay buffer (50 mM PBS with BSA (1% *w*/*v*), Tween-20 (0.1% *w*/*v*) and bronidox (0.36% *v*/*v*), 8 g/L NaCl, pH 7.4) to a concentration at 2 ng/mL and incubated for 60 min at 20 °C with shaking (750 rpm) in darkness. Next, 25 μL/well selection peptide, assay controls, or pre-diluted serum/plasma sample (1:2) were added followed by the immediate addition of 25 μL/well of SULFO-TAG (MSD GOLD SULFO-TAG NHS-Ester Conjugation, Meso Scale Discovery, cat. no. R31AA-1) labeled monoclonal antibody diluted in assay buffer to a final concentration at 25 ng/mL and the plate was incubated for 20 h at 4 °C with shaking (300 rpm) in darkness. All incubation steps were followed by washing the plate three times in washing buffer (20 mM Tris, 50 mM NaCl, pH 7.2). Finally, 150 μL/well of MSD GOLD Read Buffer (Meso Scale Discovery, cat. no. R92TG-2) was added and the plate was read immediately within 2 min in a Sector Imager 6000 (Meso Scale Discovery). SULFO-TAG enabled light emission when electricity was applied, and the light emission data was analyzed using the MSD Discovery Workbench 4.0 software (Meso Scale Discovery, Rockville, USA). The analyte concentration was calculated using a 4-parametric curve fit model. Due to low sensitivity in the ELISA, C4G was developed as a competitive assay on the Meso Scale Discovery ECLIA platform, which has the advantage of providing better sensitivity and a broader dynamic range compared to ELISA.

### 4.2. Technical Evaluation of the C4G ECLIA

#### 4.2.1. Specificity of the C4G Assay

The specificity of the antibody toward the selection peptide (MGNTGPTGAV) was tested by including an elongated peptide (FMGNTGPTGAV), a truncated peptide (GNTGPTGAV), a non-sense selection peptide (LLARDFEKNY), and a non-sense coating peptide (LLARDFEKNY-K-biotin). To test for potential cross-reactivity to other proteins with similar sequences, three peptides with one amino acid mismatch at either position one (QGNTGPTGAV), three (MGQTGPTGAV), or four (MGNSGPTGAV) from the N-terminal were included in the specificity test. Antibody specificity was calculated as the percentage of signal inhibition of two-fold diluted peptides.

The specificity of the antibody was furthermore tested by in vitro cleavage of type IV collagen with various proteases. Recombinant type IV collagen α2 chain (MyBioSource, San Diego, CA, USA) was incubated without or with the serine proteases granzyme B (Abcam, cat. no. ab168093) or human neutrophil elastase (Abcam, cat. no. Ab91099) in the ratio 10:1 and 50:1 (10 μg type IV collagen and 1/0.2 μg protease), respectively in serine protease buffer (50 mM Tris, 150 mM NaCl, pH 7.5) and with the MMPs MMP-2 (Giotto, Sesto Fiorentino, Italy, cat. no. G04MP02C) or MMP-9 (Giotto, cat. no. G04MP09C) in the ratio 10:1 in MMP buffer (50 mM Tris-HCl, 150 nM NaCl, 10 mM CaCl_2_, 10μM ZnCl, 0.05%Brij35, pH 7.5). Digestion of carboxymethylated transferrin with each of the proteases was included as a positive control, and buffer added with each protease alone was included as a negative control. The solutions were incubated at 37 °C for 1, 4, 24, or 72 h and then stored at −80 °C until analysis. The activity of proteases was confirmed by gel electrophoresis and Coomassie blue staining.

#### 4.2.2. Technical Validation

The intra- and inter-assay variation were determined by ten independent runs of seven samples in duplicates with concentrations covering the entire linear range of the standard curve. The samples consisted of four samples with different amounts of selection peptide in assay buffer and three different healthy human serum samples. Intra-assay variation was calculated as the mean coefficient of variance (CV%) within plates, and the inter-assay variation was calculated as the mean CV% between the ten plates. To determine the linearity of the assay, two-fold dilutions of human serum (*n* = 3) or EDTA plasma samples (*n* = 3) were performed, and the linearity was calculated as percentage recovery of the undiluted sample. Accuracy was determined by spiking human serum into human serum (*n* = 2), and the spiking recovery was calculated as the percentage recovery of the expected concentration (serum and serum combined). Analyte stability was tested by four repeated freeze/thaw cycles of human serum (*n* = 3 at each cycle), and the analyte recovery was calculated with the first cycle as a reference. Analyte stability was furthermore tested by incubated human serum samples (*n* = 3 at each time point) at 4 °C or 20 °C for 24 or 48 h, and recovery was calculated with samples stored at −20 °C as reference. Interference was tested by adding a low/high content of biotin (3.0/9.0 ng/mL), lipemia (1.5/5.0 mg/mL), and hemoglobin (2.5/5.0 mg/mL) to a serum sample, and the recovery was calculated with the serum sample as reference.

### 4.3. Clinical Evaluation

#### 4.3.1. Patient Samples

Serum samples were collected at baseline from stage IV melanoma patients (*n* = 54) treated with ipilimumab (3 mg/kg body weight) as the standard of care at Copenhagen University Hospital, Herlev and Aarhus University Hospital, Denmark, after informed consent and approval by the Ethics Committee for the Capital Region of Denmark (H-2-2012-058) in compliance with the Helsinki Declaration of 1975. The patients received at least two doses and maximal four doses of ipilimumab with three weeks apart. Patient exclusion and inclusion criteria are previously described [26]. Patients were examined within four weeks before the first treatment and every third month thereafter until progression. Clinical response was evaluated according to RECIST. Baseline patient characteristics are shown in Table 2.

#### 4.3.2. Biomarker Measurements

C4G was measured in duplicates in serum from the metastatic melanoma patients with the newly developed C4G ECLIA. To combine C4G levels with the degree of collagen deposition in the tumor microenvironment, previously measured PRO-C3 levels were evaluated [13]. The PRO-C3 competitive ELISA is a well-characterized assay based on a monoclonal antibody specific toward the N-proteinase cleavage site of the N-terminal pro-peptide of type III collagen and therefore measures the formation of type III collagen [35]. The ELISA is manufactured by Nordic Bioscience (Herlev, Denmark) and measurements were performed according to the manufacturer’s specifications [35].

#### 4.3.3. Statistical Analyses

C4G levels in metastatic melanoma patients with CR and PR were compared to C4G levels in patients with SD and PD with the Mann–Whitney test. The ORR in patients with low C4G levels (≤25th percentile) was compared to patients with high C4G levels (>25th percentile) with Fisher’s exact test. Kaplan–Meier survival curves were used to analyze OS for each group of patients, and a log-rank test was used to determine differences between the curves. The same tests were used to evaluate response in patients with both high C4G levels (>25th percentile) and low PRO-C3 levels (≤75th percentile) compared to patients with low C4G and/or high PRO-C3 levels. Univariate Cox proportional-hazards analysis was used to calculate HRs with 95% CIs for prediction of OS for C4G alone and in combination with PRO-C3 as well for the clinical covariates age, LDH, and prior systematic treatment. Multivariate Cox regression was used to assess the independent predictive potential of the biomarkers when adjusted for age, LDH, and prior systematic treatment. C4G was dichotomized by the 25th percentile cut-point, PRO-C3 was dichotomized by the 75th percentile cut-point and LDH was dichotomized at the 250 IU/L value. Graphs and statistical analyses were performed using MedCalc (v16.8.4) and GraphPad Prism version 7 (GraphPad Software, San Diego, CA, USA). A *p*-value of *p* < 0.05 was considered statistically significant.

## 5. Conclusions

Our findings show that a biomarker measuring granzyme B degraded type IV collagen (C4G) is predictive for response to anti-CTLA-4 therapy in metastatic melanoma patients. When combining high C4G with low PRO-C3 (tumor fibrosis) patients with this special phenotype have an even better chance of responding compared to high C4G levels alone. If validated, these collagen remodeling biomarkers may provide a non-invasive precision medicine tool for patient selection and therapeutic decision-making in the future.

## 6. Patents

A patent for the C4G assay is filed and owned by Nordic Bioscience.

## Figures and Tables

**Table 1:** Technical validation of the C4G assay.

Technical Validation Step	Results
LLMR (uncorrected for pre-dilution)	2.3 ng/mL
ULMR (uncorrected for pre-dilution)	788 ng/mL
Calc. midpoint (IC50)	8.8 ng/mL
Intra-assay variation	6%
Inter-assay variation	8%
Dilution recovery of serum	94%
Dilution recovery of EDTA plasma	106%
Spiking recovery of serum in serum	102%
Analyte recovery, 4 freeze/thaw cycles	96%
Analyte recovery 24h, 4 °C/20 °C	109%/106%
Analyte recovery 48h, 4 °C/20 °C	122%/109%
Interference test	
Biotin recovery, low/high	94%/71%
Lipemia recovery, low/high	111%/102%
Hemoglobin recovery, low/high	99%/92%

LLMR: lower limit of measurement range; ULMR: upper limit of measurement range. Percentages are reported as mean.

**Table 2:** Patient characteristics.

Variable	Value
Age at Baseline (Median with Range)	68 (35–83)
Gender (% females)	31/54 (57%)
Prior systemic therapy:	
None	30 (55%)
IFN/IL-2	18 (33%)
Temozolomide	3 (6%)
Temozolomide/Vemurafenib	1 (2%)
Vemurafenib	2 (4%)
RECIST response:	
CR	1 (2%)
PR	7 (13%)
SD	11 (20%)
PD	35 (65%)
Lactate dehydrogenase (LDH):	
≥250 IU/L	11 (21%)
<250 IU/L	42 (79%)

CR: complete response; PR: partial response; SD: stable disease; PD: progressive disease.

**Table 3:** Association between biomarkers at baseline, clinical covariates, and overall survival for metastatic melanoma patients.

Variable	Cut-Point	HR	95%CI	*p*-Value
C4G				
Univariate	>25th percentile vs. ≤25th percentile	0.48	0.24–0.98	0.045
Multivariate	>25th percentile vs. ≤25th percentile	0.43	0.19–1.00	0.051
C4G and PRO-C3				
Univariate	C4G >25th percentile and PRO-C3 ≤75th percentile vs. C4G ≤25th percentile and/or PRO-C3 >75th percentile	0.30	0.15–0.60	0.0006
Multivariate	C4G >25th percentile and PRO-C3 ≤75th percentile vs. C4G ≤25th percentile and/or PRO-C3 >75th percentile	0.35	0.18–0.72	0.004
Age at baseline	Continuous	1.02	1.00–1.05	0.084
LDH at sampling	Continuous	1.00	1.00–1.01	<0.0001
	High (≥250 IU/L) vs. low (<250 IU/L)	2.50	1.20–5.21	0.015
Prior systematic therapy		1.38	0.72–2.63	0.333

Hazard ratios (HR) were calculated by Cox proportional-hazards analysis. By the univariate Cox regression, C4G was dichotomized by the 25th percentile cut-point with high levels (14.6–78.6 ng/mL) used as a reference to calculate the HR for patients with low levels (7.9–14.5 ng/mL). PRO-C3 was dichotomized by the 75th percentile cut-point (≤75th percentile: 5.7–17.5 ng/mL and >75th percentile: 20.7–113.3 ng/mL). By the multivariate Cox regression, the biomarkers were adjusted for age, lactate dehydrogenase (LDH), and prior systematic treatment.

## Supplementary Materials

The following are available online at https://www.mdpi.com/2072-6694/12/10/2786/s1, Figure S1: Serum titration was performed to monitor the immune response of the mice. The antibody’s reactivity in a competitive ELISA was tested towards the selection peptide (MGNTGPTGAV), an elongated peptide (FMGNTGPTGAV), the deselections peptide 1 (MGQTGPTGAV), 2 (MGNSGPTGAV), and 3 (QGNTGPTGAV), and the immunogen (MGNTGPTGAV-KLH), Figure S2: Characterization of the monoclonal antibody from hybridoma cells after fusion. The monoclonal antibody’s reactivity in a competitive ELISA was tested towards the selection peptide (MGNTGPTGAV), a truncated peptide (GNTGPTGAV), an elongated peptide (FMGNTGPTGAV), the immunogen (MGNTGPTGAV-KLH), and the deselections peptide 1 (MGQTGPTGAV), 2 (MGNSGPTGAV), and 3 (QGNTGPTGAV).
