# Supplementary material for: Granzyme B Degraded Type IV Collagen Products in Serum Identify Melanoma Patients Responding to Immune Checkpoint Blockade
Source: Cancers (Basel). 2020 Sep 28;12(10):2786. doi: 10.3390/cancers12102786 (PMC7601429; doi:10.3390/cancers12102786)
Supplement: Supplementary file 1 [file cancers-12-02786-s001.pdf]

Supplementary Material: Granzyme B Degraded Type IV Collagen Products in Serum Identify Melanoma Patients Responding to Immune Checkpoint Blockade

Christina Jensen, Dovile Sinkeviciute, Daniel Hargbøl Madsen, Patrik Önnerfjord, Morten Hansen, Henrik Schmidt, Morten Asser Karsdal, Inge Marie Svane and Nicholas Willumsen

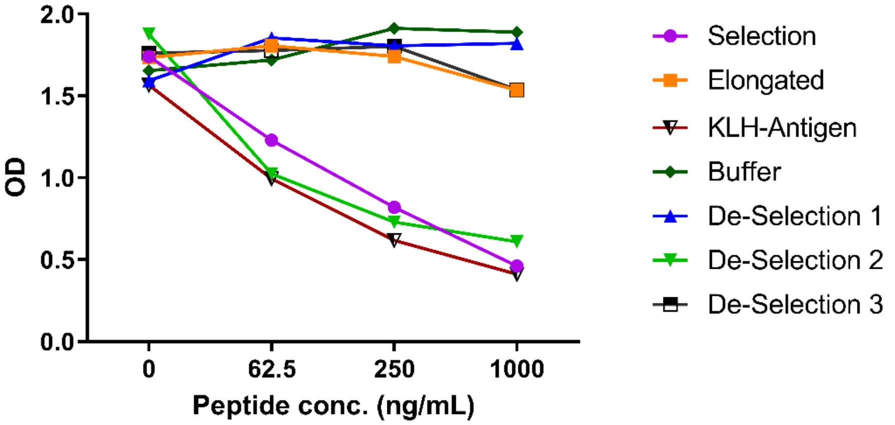

Figure S1. Serum titration was performed to monitor the immune response of the mice. The antibody's reactivity in a competitive ELISA was tested towards the selection peptide (MGNTGPTGAV), an elongated peptide (FMGNTGPTGAV), the deselections peptide 1 (MGQTGPTGAV), 2 (MGNSGPTGAV), and 3 (QGNTGPTGAV), and the immunogen (MGNTGPTGAV-KLH).

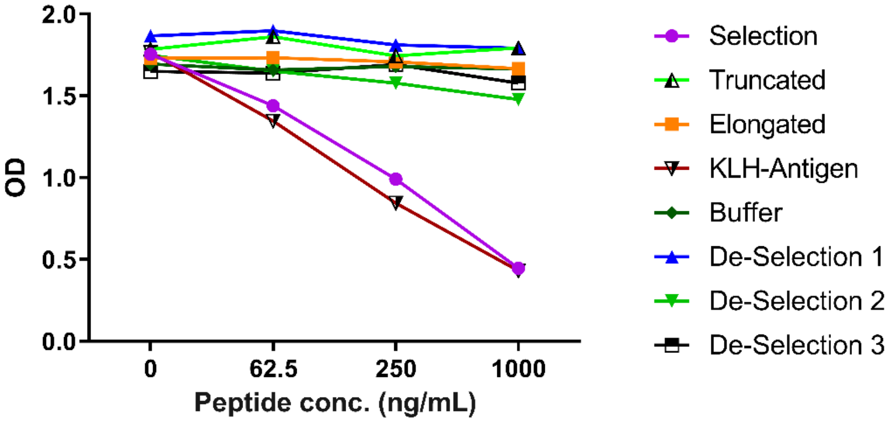

Figure S2. Characterization of the monoclonal antibody from hybridoma cells after fusion. The monoclonal antibody's reactivity in a competitive ELISA was tested towards the selection peptide (MGNTGPTGAV), a truncated peptide (GNTGPTGAV), an elongated peptide (FMGNTGPTGAV), the immunogen (MGNTGPTGAV-KLH), and the deselections peptide 1 (MGQTGPTGAV), 2 (MGNSGPTGAV), and 3 (QGNTGPTGAV).

© 2020 by the authors. Licensee MDPI, Basel, Switzerland. This article is an open access article distributed under the terms and conditions of the Creative Commons Attribution (CC BY) license (http://creativecommons.org/licenses/by/4.0/).
